# Bis(μ_2_-benzoato-κ^2^ *O*:*O*′)bis­[(benzoato-κ^2^ *O*,*O*′)bis(4,4′-bi­pyridine-κ*N*)cobalt(II)]–benzoic acid (1/6)

**DOI:** 10.1107/S1600536813033357

**Published:** 2013-12-18

**Authors:** Rodolfo Peña-Rodríguez, José María Rivera, Raúl Colorado-Peralta, Angélica María Duarte-Hernández, Angelina Flores-Parra

**Affiliations:** a Facultad de Ciencias Químicas, Universidad Veracruzana, Prolongación Oriente 6, No. 1009, Colonia Rafael Alvarado, Apartado Postal 215, CP 94340, Orizaba, Veracruz, Mexico; b Departamento de Química, Centro de Investigación y de Estudios Avanzados del, Instituto Politécnico Nacional, CP 07360, México, DF, Mexico

## Abstract

In the title compound, [Co_2_(C_7_H_5_O_2_)_4_(C_10_H_8_N_2_)_4_]·6C_6_H_5_COOH, the centrosymmetric cobalt dimer co-crystallizes with six mol­ecules of benzoic acid. Each Co^II^ atom is coordinated by four O atoms in a distorted square-planar arrangement while the N atoms are located in apical positions. The dihedral angles between the rings comprising each of the 4,4′-bipyridyl ligands are 25.2 (2) and 22.8 (2)°. In the crystal, the three-dimensional network is assembled by O—H⋯O and C—H⋯O hydrogen bonds.

## Related literature   

For polymer structures with benzoate and 4,4′-bipyridyl ligands coordinated to cobalt(II), see: Song *et al.* (2009 ▶), Zhang *et al.* (2007 ▶); to copper(II), see: Wu *et al.* (2007 ▶); to cadmium(II) and zinc(II), see: Murugesapandian & Roesky (2011*a* ▶,*b* ▶).
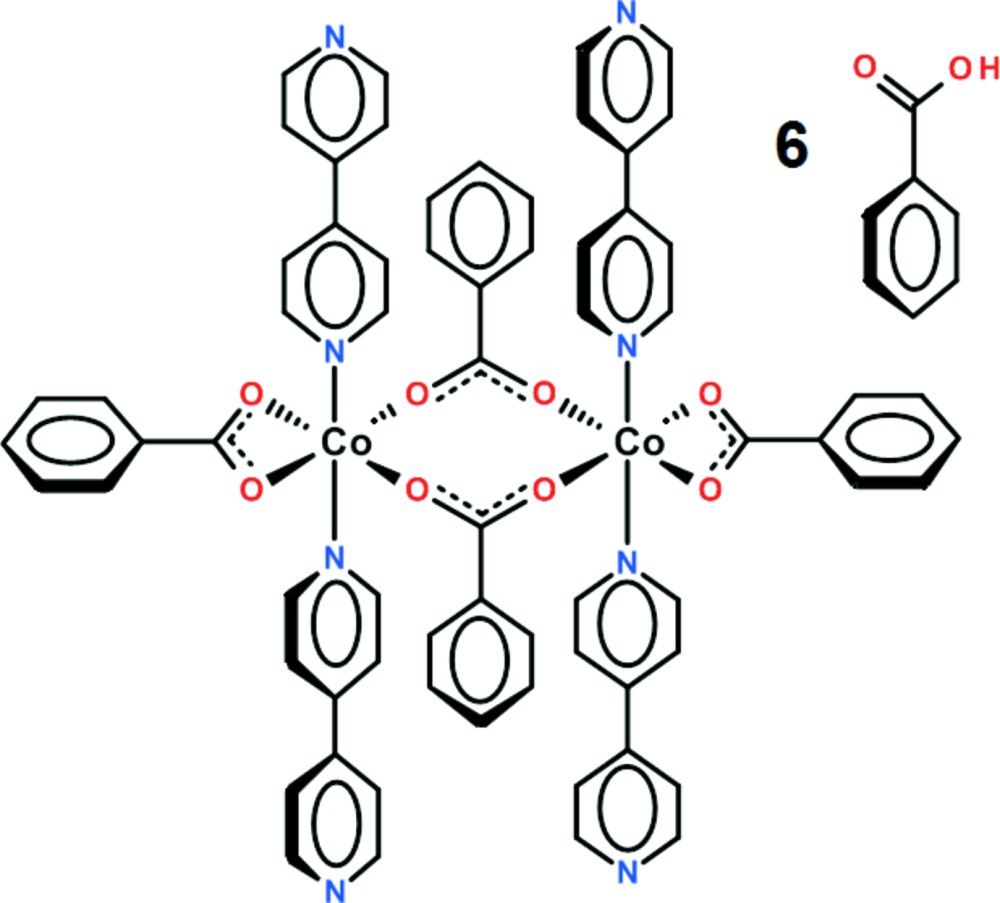


## Experimental   

### 

#### Crystal data  

[Co_2_(C_7_H_5_O_2_)_4_(C_10_H_8_N_2_)_4_]·6C_7_H_6_O_2_
*M* *_r_* = 1959.74
Triclinic, 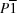

*a* = 10.4977 (6) Å
*b* = 15.8329 (7) Å
*c* = 16.3994 (8) Å
α = 64.222 (3)°
β = 87.792 (3)°
γ = 86.469 (3)°
*V* = 2449.6 (2) Å^3^
*Z* = 1
Mo *K*α radiation
μ = 0.41 mm^−1^
*T* = 293 K
0.2 × 0.1 × 0.05 mm

#### Data collection  

Nonius KappaCCD diffractometer
Absorption correction: multi-scan (*SORTAV*; Blessing, 1987 ▶, 1989 ▶, 1995 ▶) *T*_min_ = 0.925, *T*_max_ = 0.980
28546 measured reflections
11064 independent reflections
5118 reflections with *I* > 2σ(*I*)
*R*_int_ = 0.082

#### Refinement  

*R*[*F* ^2^ > 2σ(*F* ^2^)] = 0.076
*wR*(*F* ^2^) = 0.157
*S* = 1.03
11064 reflections
631 parameters
H-atom parameters constrained
Δρ_max_ = 0.32 e Å^−3^
Δρ_min_ = −0.31 e Å^−3^

### 

Data collection: *COLLECT* (Bruker, 2004 ▶); cell refinement: *SCALEPACK* (Otwinowski & Minor, 1997 ▶); data reduction: *DENZO* (Otwinowski & Minor, 1997 ▶) and *SCALEPACK*; program(s) used to solve structure: *SHELXS97* (Sheldrick, 2008 ▶); program(s) used to refine structure: *SHELXL97* (Sheldrick, 2008 ▶) ▶; molecular graphics: *Mercury* (Macrae *et al.*, 2006 ▶); software used to prepare material for publication: *WinGX* (Farrugia, 2012 ▶), *enCIFer* (Allen, 2004 ▶) and *publCIF* (Westrip, 2010 ▶).

## Supplementary Material

Crystal structure: contains datablock(s) global, I. DOI: 10.1107/S1600536813033357/mw2119sup1.cif

Structure factors: contains datablock(s) I. DOI: 10.1107/S1600536813033357/mw2119Isup2.hkl

Additional supporting information:  crystallographic information; 3D view; checkCIF report

## Figures and Tables

**Table 1:** Hydrogen-bond geometry (Å, °)

*D*—H⋯*A*	*D*—H	H⋯*A*	*D*⋯*A*	*D*—H⋯*A*
O6—H6*A*⋯N2^i^	0.82	1.91	2.726 (5)	177
O7—H7*A*⋯O1^ii^	0.82	1.86	2.656 (4)	164
O9—H9*A*⋯N5^iii^	0.82	1.91	2.732 (5)	176

Symmetry codes: (i) 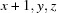; (ii) 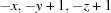; (iii) 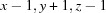.

**Fig. 1.**
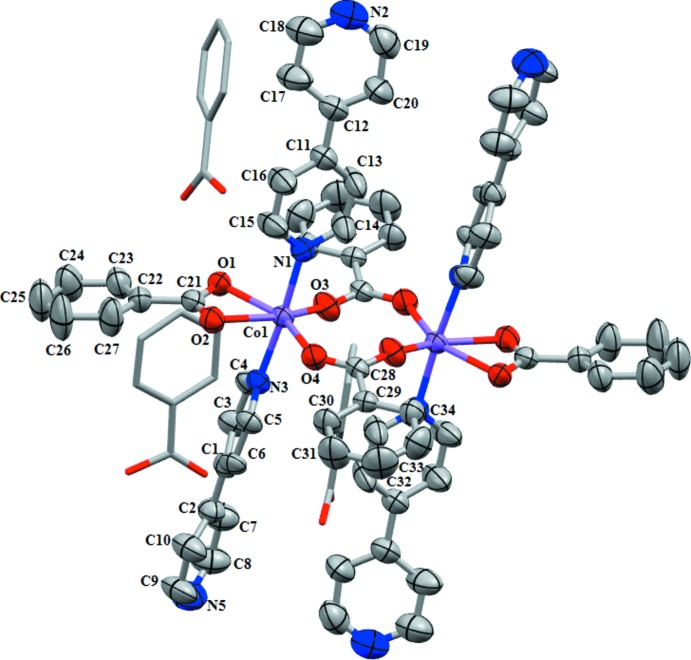
The molecular structure of the title compound, showing the atom-labelling scheme. Displacement ellipsoids are drawn at the 50% probability level, three benzoic acid molecules are represented by sticks and H atoms are omitted for clarity.

**Fig. 2.**
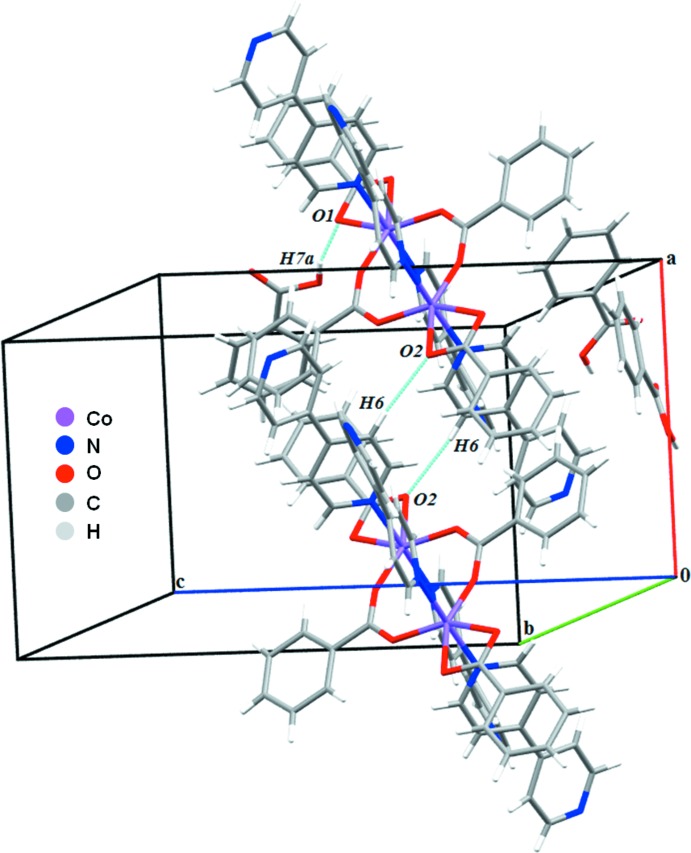
Crystal packing of the title compound viewed along b axis. Intermolecular hydrogen bonds are shown as blue dashed lines.

## supplementary crystallographic information

### 1. Comment

The design of metal-organic coordination compounds is of current interest in the fields of supramolecular chemistry and crystal engineering. This interest stems from their potential applications as functional materials, such as in gas storage, ion-exchange, catalysis, magnetism and molecular sensing (Song *et al.*, 2009; Zhang *et al.*, 2007). 4,4'-Bipyridine is an excellent, rigid bridging ligand for the construction of novel metal-organic coordination compounds due to its various coordinative modes with metal ions (Wu *et al.*, 2007; Murugesapandian & Roesky, 2011*a*, 2011*b*). Currently all the metal-organic coordination compounds obtained with benzoic acid and 4,4'-bipyridine are polymeric. The title compound is the first example of a dimeric species with these two ligands.

The title compound is a cobalt(II) dimer having crystallographically-imposed centrosymmetry (Fig. 1) together with six molecules of benzoic acid in the lattice. Each cobalt(II) ion displays a distorted octahedral coordination geometry being surrounded by four oxygen atoms of one chelating and two bridging benzoate anions and two *trans*-disposed, monodentate 4,4'-bipyridine ligands. In the central portion of the dimer, the two metal ions and the two bidentate bridging benzoate ions form an eight-membered ring. The dihedral angles between the rings of the 4,4'-bipyridine ligands are 25.2 (2)° (ligand containing N1 and N2) and 22.8 (2)° (ligand containing N3 and N5). In the crystal structure weak O—H···O and C—H···O hydrogen bonds link the molecules into an infinite one-dimensional chain extending along the *b* axis [O7—H7a···O1,1.859 Å, 164.10°; C6—H6···O2, 2.366 Å, 167.54°]. (Fig. 2).

### 2. Experimental

A solution of cobalt(II) nitrate hexahydrate (93.1 mg 0.32 mmol) in 5 mL of deionized water was added dropwise to 5 mL of a methanol solution of benzoic anhydride (72.4 mg 0.32 mmol). The reaction mixture was refluxed at 80 °C without stirring after which a solution of 4,4'-bipyridine (50 mg 0.32 mmol) in 10 mL of methanol was slowly added at room temperature. The solid was crystallized from solution giving red crystals of the title compound which were suitablefor X-ray crystal structure analysis and fully characterized by standard analytical methods. *M.p.* > 350°C.

### 3. Refinement

H atoms were positioned geometrically and refined using a riding model approximation with distance O—H = 0.82 Å and C—H = 0.93 Å, with *U*_iso_(H) = 1.2*U*_eq_(C,*O*).

### Crystal data

**Table:**

[Co_2_(C_7_H_5_O_2_)_4_(C_10_H_8_N_2_)_4_]·6C_7_H_6_O_2_	*Z* = 1
*M**_r_* = 1959.74	*F*(000) = 1018
Triclinic, *P*1	*D*_x_ = 1.329 Mg m^−^^3^
Hall symbol: -P 1	Melting point: 350 K
*a* = 10.4977 (6) Å	Mo *K*α radiation, λ = 0.71073 Å
*b* = 15.8329 (7) Å	Cell parameters from 28061 reflections
*c* = 16.3994 (8) Å	θ = 1.0–27.5°
α = 64.222 (3)°	µ = 0.41 mm^−^^1^
β = 87.792 (3)°	*T* = 293 K
γ = 86.469 (3)°	Prism, red
*V* = 2449.6 (2) Å^3^	0.2 × 0.1 × 0.05 mm

### Data collection

**Table:**

Nonius KappaCCD diffractometer	11064 independent reflections
Radiation source: Enraf–Nonius FR590	5118 reflections with *I* > 2σ(*I*)
Graphite monochromator	*R*_int_ = 0.082
Detector resolution: 9 pixels mm^-1^	θ_max_ = 27.4°, θ_min_ = 2.4°
CCD rotation images, thick slices scans	*h* = −13→12
Absorption correction: multi-scan (*SORTAV*; Blessing, 1987, 1989, 1995)	*k* = −20→20
*T*_min_ = 0.925, *T*_max_ = 0.980	*l* = −20→21
28546 measured reflections	

### Refinement

**Table:**

Refinement on *F*^2^	Primary atom site location: structure-invariant direct methods
Least-squares matrix: full	Secondary atom site location: difference Fourier map
*R*[*F*^2^ > 2σ(*F*^2^)] = 0.076	Hydrogen site location: inferred from neighbouring sites
*wR*(*F*^2^) = 0.157	H-atom parameters constrained
*S* = 1.03	*w* = 1/[σ^2^(*F*_o_^2^) + (0.0388*P*)^2^ + 1.8382*P*] where *P* = (*F*_o_^2^ + 2*F*_c_^2^)/3
11064 reflections	(Δ/σ)_max_ = 0.001
631 parameters	Δρ_max_ = 0.32 e Å^−^^3^
0 restraints	Δρ_min_ = −0.31 e Å^−^^3^

### Special details

**Table:**

Geometry. All e.s.d.'s (except the e.s.d. in the dihedral angle between two l.s. planes) are estimated using the full covariance matrix. The cell e.s.d.'s are taken into account individually in the estimation of e.s.d.'s in distances, angles and torsion angles; correlations between e.s.d.'s in cell parameters are only used when they are defined by crystal symmetry. An approximate (isotropic) treatment of cell e.s.d.'s is used for estimating e.s.d.'s involving l.s. planes.
Refinement. Refinement of *F*^2^ against ALL reflections. The weighted *R*-factor *wR* and goodness of fit *S* are based on *F*^2^, conventional *R*-factors *R* are based on *F*, with *F* set to zero for negative *F*^2^. The threshold expression of *F*^2^ > σ(*F*^2^) is used only for calculating *R*-factors(gt) *etc*. and is not relevant to the choice of reflections for refinement. *R*-factors based on *F*^2^ are statistically about twice as large as those based on *F*, and *R*-factors based on ALL data will be even larger.

### Fractional atomic coordinates and isotropic or equivalent isotropic displacement parameters (Å^2^)

**Table:**

	*x*	*y*	*z*	*U*_iso_*/*U*_eq_	
Co1	0.13907 (4)	0.09130 (3)	0.51110 (3)	0.04482 (16)	
O1	0.1879 (2)	0.16963 (17)	0.58558 (17)	0.0566 (7)	
O2	0.3130 (2)	0.17380 (17)	0.47431 (17)	0.0573 (7)	
O3	−0.0072 (2)	0.02611 (18)	0.59081 (16)	0.0605 (7)	
O4	0.1804 (2)	0.04234 (18)	0.41807 (16)	0.0563 (7)	
N1	0.0204 (3)	0.2073 (2)	0.4218 (2)	0.0511 (8)	
N2	−0.3787 (5)	0.6089 (3)	0.1719 (3)	0.0927 (13)	
N3	0.2633 (3)	−0.0192 (2)	0.60462 (19)	0.0477 (7)	
N5	0.6503 (5)	−0.3951 (3)	0.9135 (3)	0.0887 (12)	
C1	0.4254 (3)	−0.1614 (2)	0.7278 (2)	0.0517 (9)	
C2	0.5065 (4)	−0.2405 (3)	0.7921 (3)	0.0583 (10)	
C3	0.3224 (4)	−0.1222 (3)	0.7567 (3)	0.0636 (11)	
H3	0.3052	−0.1427	0.8182	0.076*	
C4	0.2452 (4)	−0.0522 (3)	0.6933 (3)	0.0602 (10)	
H4	0.1765	−0.0268	0.7143	0.072*	
C5	0.3647 (3)	−0.0556 (2)	0.5767 (2)	0.0490 (9)	
H5	0.3806	−0.0327	0.5148	0.059*	
C6	0.4468 (3)	−0.1251 (2)	0.6347 (2)	0.0516 (9)	
H6	0.5162	−0.1478	0.6118	0.062*	
C7	0.4598 (5)	−0.2980 (3)	0.8773 (3)	0.0776 (13)	
H7	0.3786	−0.2856	0.8956	0.093*	
C8	0.5339 (6)	−0.3730 (3)	0.9344 (3)	0.0919 (16)	
H8	0.5007	−0.4106	0.9913	0.11*	
C9	0.6958 (5)	−0.3408 (4)	0.8324 (4)	0.0938 (16)	
H9	0.777	−0.3556	0.8161	0.113*	
C10	0.6279 (4)	−0.2627 (3)	0.7704 (3)	0.0837 (14)	
H10	0.6642	−0.2254	0.7145	0.1*	
C11	−0.1404 (4)	0.3636 (3)	0.3194 (2)	0.0538 (10)	
C12	−0.2251 (4)	0.4474 (3)	0.2685 (3)	0.0605 (10)	
C13	−0.1842 (4)	0.2761 (3)	0.3651 (3)	0.0786 (14)	
H13	−0.2705	0.2668	0.3634	0.094*	
C14	−0.1030 (4)	0.2013 (3)	0.4138 (3)	0.0778 (14)	
H14	−0.1368	0.1424	0.4431	0.093*	
C15	0.0627 (4)	0.2928 (3)	0.3749 (3)	0.0853 (15)	
H15	0.1494	0.3005	0.377	0.102*	
C16	−0.0126 (4)	0.3702 (3)	0.3238 (3)	0.0846 (15)	
H16	0.0239	0.4278	0.2918	0.101*	
C17	−0.1911 (5)	0.5364 (3)	0.2540 (3)	0.0911 (16)	
H17	−0.1145	0.5437	0.2763	0.109*	
C18	−0.2705 (6)	0.6136 (4)	0.2066 (4)	0.1049 (18)	
H18	−0.2462	0.6722	0.1988	0.126*	
C19	−0.4141 (5)	0.5272 (5)	0.1853 (3)	0.0924 (16)	
H19	−0.4916	0.5231	0.1619	0.111*	
C20	−0.3392 (5)	0.4428 (4)	0.2345 (3)	0.0889 (15)	
H20	−0.3682	0.3851	0.2433	0.107*	
C21	0.2930 (3)	0.1908 (2)	0.5415 (3)	0.0523 (9)	
C22	0.3932 (4)	0.2327 (3)	0.5724 (3)	0.0592 (10)	
C23	0.3760 (4)	0.2431 (3)	0.6515 (3)	0.0746 (12)	
H23	0.3006	0.2258	0.6849	0.089*	
C24	0.4712 (5)	0.2794 (4)	0.6811 (4)	0.1020 (17)	
H24	0.4595	0.2866	0.7342	0.122*	
C25	0.5817 (6)	0.3044 (4)	0.6327 (5)	0.116 (2)	
H25	0.6454	0.3289	0.6527	0.14*	
C26	0.5993 (5)	0.2940 (5)	0.5558 (5)	0.124 (2)	
H26	0.6753	0.3112	0.5231	0.149*	
C27	0.5054 (4)	0.2580 (4)	0.5248 (4)	0.0937 (16)	
H27	0.5186	0.2509	0.4716	0.112*	
C28	−0.1194 (3)	0.0033 (2)	0.6124 (2)	0.0420 (8)	
C29	−0.1875 (3)	0.0308 (2)	0.6794 (2)	0.0431 (8)	
C30	−0.3148 (4)	0.0119 (3)	0.7003 (3)	0.0595 (10)	
H30	−0.3578	−0.0171	0.6716	0.071*	
C31	−0.3783 (4)	0.0354 (4)	0.7630 (3)	0.0842 (14)	
H31	−0.464	0.023	0.7763	0.101*	
C32	−0.3144 (6)	0.0772 (4)	0.8054 (3)	0.0948 (16)	
H32	−0.3569	0.0923	0.8484	0.114*	
C33	−0.1893 (5)	0.0970 (3)	0.7856 (3)	0.0824 (14)	
H33	−0.1472	0.1257	0.815	0.099*	
C34	−0.1249 (4)	0.0747 (3)	0.7221 (3)	0.0609 (10)	
H34	−0.0399	0.0891	0.7079	0.073*	
O5	0.3391 (4)	0.6953 (3)	0.0736 (3)	0.1194 (14)	
O6	0.5022 (4)	0.7786 (2)	0.0694 (2)	0.1021 (11)	
H6A	0.5358	0.7265	0.0994	0.153*	
C42	0.3856 (6)	0.7699 (4)	0.0509 (3)	0.0842 (14)	
C43	0.3183 (4)	0.8629 (3)	−0.0038 (3)	0.0627 (11)	
C44	0.1891 (5)	0.8649 (4)	−0.0154 (3)	0.0866 (14)	
H44	0.146	0.8094	0.0106	0.104*	
C45	0.1230 (5)	0.9484 (5)	−0.0651 (4)	0.1014 (17)	
H45	0.0355	0.9495	−0.0725	0.122*	
C46	0.1856 (6)	1.0296 (4)	−0.1035 (4)	0.1030 (18)	
H46	0.1408	1.0863	−0.1365	0.124*	
C47	0.3141 (6)	1.0277 (3)	−0.0935 (3)	0.0933 (16)	
H47	0.3571	1.0831	−0.1208	0.112*	
C48	0.3802 (5)	0.9451 (3)	−0.0436 (3)	0.0749 (12)	
H48	0.4678	0.9446	−0.0366	0.09*	
O7	−0.0189 (3)	0.6991 (2)	0.4128 (2)	0.0877 (9)	
H7A	−0.0735	0.7415	0.4032	0.132*	
O8	−0.0534 (3)	0.7343 (2)	0.2686 (3)	0.0932 (10)	
C49	0.0020 (4)	0.6876 (3)	0.3376 (4)	0.0683 (12)	
C50	0.1003 (4)	0.6121 (3)	0.3495 (3)	0.0649 (11)	
C51	0.1503 (4)	0.6056 (3)	0.2726 (4)	0.0782 (13)	
H51	0.1215	0.6478	0.2156	0.094*	
C52	0.2422 (5)	0.5369 (4)	0.2805 (5)	0.0965 (16)	
H52	0.2754	0.5326	0.229	0.116*	
C53	0.2843 (6)	0.4755 (4)	0.3636 (6)	0.1079 (19)	
H53	0.3467	0.4292	0.3685	0.129*	
C54	0.2367 (5)	0.4804 (4)	0.4403 (5)	0.1045 (18)	
H54	0.2661	0.4377	0.4969	0.125*	
C55	0.1436 (5)	0.5500 (3)	0.4328 (4)	0.0834 (14)	
H55	0.1109	0.5541	0.4846	0.1*	
O9	−0.2055 (3)	0.4497 (2)	0.0229 (2)	0.0972 (10)	
H9A	−0.2473	0.4977	−0.0088	0.146*	
O10	−0.0947 (3)	0.4821 (2)	−0.1046 (2)	0.1078 (12)	
C35	−0.1129 (5)	0.4338 (3)	−0.0259 (4)	0.0774 (13)	
C36	−0.0322 (4)	0.3462 (3)	0.0283 (3)	0.0711 (12)	
C37	0.0617 (5)	0.3194 (4)	−0.0164 (4)	0.1078 (19)	
H37	0.0745	0.3556	−0.0781	0.129*	
C38	0.1384 (6)	0.2389 (4)	0.0287 (4)	0.118 (2)	
H38	0.2014	0.2205	−0.0026	0.141*	
C39	0.1209 (6)	0.1874 (4)	0.1187 (4)	0.1061 (18)	
H39	0.173	0.1339	0.1498	0.127*	
C40	0.0265 (6)	0.2138 (4)	0.1645 (4)	0.1014 (17)	
H40	0.0138	0.178	0.2262	0.122*	
C41	−0.0491 (5)	0.2937 (3)	0.1181 (3)	0.0863 (14)	
H41	−0.1128	0.3119	0.1491	0.104*	

### Atomic displacement parameters (Å^2^)

**Table:**

	*U*^11^	*U*^22^	*U*^33^	*U*^12^	*U*^13^	*U*^23^
Co1	0.0362 (3)	0.0504 (3)	0.0510 (3)	−0.0013 (2)	0.0021 (2)	−0.0252 (2)
O1	0.0409 (15)	0.0625 (16)	0.0784 (18)	−0.0047 (12)	0.0067 (13)	−0.0421 (14)
O2	0.0486 (16)	0.0696 (17)	0.0632 (17)	−0.0057 (13)	0.0042 (13)	−0.0378 (15)
O3	0.0380 (15)	0.0825 (18)	0.0580 (16)	−0.0074 (13)	0.0108 (12)	−0.0280 (14)
O4	0.0479 (16)	0.0741 (17)	0.0585 (16)	−0.0023 (13)	0.0033 (12)	−0.0400 (14)
N1	0.0431 (19)	0.0514 (19)	0.0581 (19)	−0.0026 (15)	0.0005 (15)	−0.0230 (16)
N2	0.089 (3)	0.100 (3)	0.078 (3)	0.026 (3)	−0.013 (2)	−0.031 (3)
N3	0.0403 (18)	0.0574 (18)	0.0470 (19)	−0.0008 (14)	0.0029 (14)	−0.0246 (15)
N5	0.101 (3)	0.070 (3)	0.077 (3)	0.016 (2)	−0.021 (3)	−0.017 (2)
C1	0.043 (2)	0.055 (2)	0.055 (2)	−0.0056 (18)	0.0011 (18)	−0.0213 (19)
C2	0.055 (3)	0.051 (2)	0.063 (3)	0.0023 (19)	−0.008 (2)	−0.019 (2)
C3	0.061 (3)	0.077 (3)	0.048 (2)	0.011 (2)	0.002 (2)	−0.025 (2)
C4	0.049 (2)	0.075 (3)	0.060 (3)	0.008 (2)	0.005 (2)	−0.034 (2)
C5	0.037 (2)	0.058 (2)	0.048 (2)	−0.0008 (17)	0.0086 (17)	−0.0211 (19)
C6	0.033 (2)	0.057 (2)	0.062 (2)	0.0048 (17)	0.0045 (18)	−0.024 (2)
C7	0.084 (3)	0.073 (3)	0.063 (3)	0.014 (3)	0.000 (2)	−0.019 (2)
C8	0.108 (5)	0.081 (3)	0.068 (3)	0.018 (3)	0.001 (3)	−0.018 (3)
C9	0.059 (3)	0.088 (3)	0.109 (4)	0.018 (3)	−0.005 (3)	−0.022 (3)
C10	0.061 (3)	0.079 (3)	0.085 (3)	0.014 (2)	−0.005 (3)	−0.014 (3)
C11	0.046 (2)	0.059 (2)	0.053 (2)	0.0037 (19)	−0.0004 (18)	−0.021 (2)
C12	0.051 (3)	0.067 (3)	0.061 (3)	0.009 (2)	−0.002 (2)	−0.026 (2)
C13	0.046 (3)	0.076 (3)	0.088 (3)	−0.010 (2)	−0.021 (2)	−0.009 (3)
C14	0.060 (3)	0.060 (3)	0.091 (3)	−0.016 (2)	−0.020 (2)	−0.008 (2)
C15	0.047 (3)	0.062 (3)	0.115 (4)	−0.002 (2)	−0.004 (3)	−0.009 (3)
C16	0.054 (3)	0.055 (3)	0.115 (4)	−0.003 (2)	−0.012 (3)	−0.009 (3)
C17	0.067 (3)	0.067 (3)	0.114 (4)	0.012 (3)	−0.010 (3)	−0.016 (3)
C18	0.099 (5)	0.079 (4)	0.115 (5)	0.019 (3)	−0.006 (4)	−0.025 (3)
C19	0.072 (4)	0.125 (5)	0.089 (4)	0.013 (3)	−0.025 (3)	−0.056 (4)
C20	0.079 (4)	0.087 (3)	0.105 (4)	0.020 (3)	−0.036 (3)	−0.046 (3)
C21	0.041 (2)	0.045 (2)	0.069 (3)	0.0019 (17)	−0.003 (2)	−0.024 (2)
C22	0.047 (2)	0.064 (2)	0.076 (3)	−0.0024 (19)	−0.007 (2)	−0.038 (2)
C23	0.055 (3)	0.094 (3)	0.087 (3)	−0.008 (2)	−0.008 (2)	−0.050 (3)
C24	0.075 (4)	0.151 (5)	0.121 (4)	−0.015 (4)	−0.005 (3)	−0.096 (4)
C25	0.078 (4)	0.158 (6)	0.155 (6)	−0.038 (4)	−0.005 (4)	−0.103 (5)
C26	0.077 (4)	0.186 (6)	0.151 (6)	−0.068 (4)	0.023 (4)	−0.105 (5)
C27	0.063 (3)	0.129 (4)	0.116 (4)	−0.042 (3)	0.024 (3)	−0.076 (4)
C28	0.034 (2)	0.0437 (19)	0.0396 (19)	0.0041 (16)	−0.0016 (16)	−0.0107 (16)
C29	0.041 (2)	0.0428 (19)	0.043 (2)	0.0038 (16)	−0.0002 (16)	−0.0167 (16)
C30	0.047 (2)	0.075 (3)	0.064 (3)	−0.005 (2)	0.012 (2)	−0.038 (2)
C31	0.058 (3)	0.124 (4)	0.091 (3)	−0.010 (3)	0.025 (3)	−0.067 (3)
C32	0.096 (4)	0.122 (4)	0.091 (4)	0.001 (3)	0.024 (3)	−0.072 (3)
C33	0.093 (4)	0.096 (4)	0.082 (3)	−0.007 (3)	−0.001 (3)	−0.061 (3)
C34	0.055 (3)	0.070 (3)	0.065 (3)	−0.004 (2)	−0.001 (2)	−0.036 (2)
O5	0.145 (4)	0.067 (2)	0.124 (3)	−0.001 (2)	0.011 (3)	−0.022 (2)
O6	0.107 (3)	0.089 (2)	0.102 (3)	0.032 (2)	−0.035 (2)	−0.035 (2)
C42	0.100 (4)	0.090 (4)	0.067 (3)	0.016 (3)	−0.004 (3)	−0.040 (3)
C43	0.070 (3)	0.067 (3)	0.050 (2)	0.017 (2)	−0.004 (2)	−0.027 (2)
C44	0.087 (4)	0.085 (3)	0.092 (4)	−0.003 (3)	0.001 (3)	−0.041 (3)
C45	0.065 (4)	0.125 (5)	0.124 (5)	0.015 (4)	−0.012 (3)	−0.065 (4)
C46	0.107 (5)	0.097 (4)	0.094 (4)	0.036 (4)	−0.027 (4)	−0.033 (3)
C47	0.101 (4)	0.073 (3)	0.092 (4)	0.001 (3)	−0.008 (3)	−0.023 (3)
C48	0.071 (3)	0.077 (3)	0.066 (3)	0.010 (3)	−0.008 (2)	−0.022 (2)
O7	0.084 (2)	0.082 (2)	0.095 (2)	0.0141 (17)	0.0120 (18)	−0.0397 (19)
O8	0.075 (2)	0.102 (2)	0.107 (3)	0.0221 (19)	−0.018 (2)	−0.050 (2)
C49	0.053 (3)	0.064 (3)	0.092 (4)	−0.006 (2)	0.009 (3)	−0.038 (3)
C50	0.047 (3)	0.056 (3)	0.091 (3)	−0.009 (2)	0.008 (2)	−0.031 (3)
C51	0.058 (3)	0.083 (3)	0.102 (4)	−0.003 (3)	0.005 (3)	−0.048 (3)
C52	0.071 (4)	0.101 (4)	0.143 (5)	−0.001 (3)	0.013 (3)	−0.079 (4)
C53	0.087 (4)	0.075 (4)	0.163 (6)	0.008 (3)	0.014 (4)	−0.056 (4)
C54	0.093 (4)	0.067 (3)	0.124 (5)	0.006 (3)	0.003 (4)	−0.015 (3)
C55	0.075 (3)	0.062 (3)	0.100 (4)	−0.001 (3)	0.017 (3)	−0.025 (3)
O9	0.095 (3)	0.089 (2)	0.091 (2)	0.0281 (19)	−0.011 (2)	−0.0262 (19)
O10	0.103 (3)	0.089 (2)	0.085 (3)	0.017 (2)	0.000 (2)	0.002 (2)
C35	0.072 (3)	0.073 (3)	0.076 (3)	0.005 (3)	−0.011 (3)	−0.022 (3)
C36	0.066 (3)	0.061 (3)	0.072 (3)	0.001 (2)	−0.009 (2)	−0.015 (2)
C37	0.095 (4)	0.099 (4)	0.087 (4)	0.018 (3)	0.011 (3)	−0.004 (3)
C38	0.099 (4)	0.104 (4)	0.113 (5)	0.033 (4)	0.011 (4)	−0.018 (4)
C39	0.093 (4)	0.080 (4)	0.109 (5)	0.016 (3)	−0.019 (4)	−0.008 (3)
C40	0.108 (5)	0.091 (4)	0.076 (3)	0.006 (3)	−0.016 (3)	−0.009 (3)
C41	0.092 (4)	0.082 (3)	0.077 (3)	0.008 (3)	−0.013 (3)	−0.028 (3)

### Geometric parameters (Å, º)

**Table:**

Co1—O3	1.999 (2)	C26—C27	1.384 (6)
Co1—O4	2.013 (2)	C26—H26	0.93
Co1—N1	2.142 (3)	C27—H27	0.93
Co1—N3	2.157 (3)	C28—O4^i^	1.257 (4)
Co1—O1	2.176 (2)	C28—C29	1.491 (5)
Co1—O2	2.220 (2)	C29—C30	1.385 (5)
O1—C21	1.275 (4)	C29—C34	1.385 (5)
O2—C21	1.250 (4)	C30—C31	1.374 (5)
O3—C28	1.247 (4)	C30—H30	0.93
O4—C28^i^	1.257 (4)	C31—C32	1.364 (6)
N1—C14	1.321 (5)	C31—H31	0.93
N1—C15	1.327 (5)	C32—C33	1.363 (6)
N2—C19	1.290 (6)	C32—H32	0.93
N2—C18	1.311 (6)	C33—C34	1.380 (6)
N3—C4	1.324 (4)	C33—H33	0.93
N3—C5	1.338 (4)	C34—H34	0.93
N5—C9	1.320 (6)	O5—C42	1.204 (6)
N5—C8	1.324 (6)	O6—C42	1.302 (6)
C1—C3	1.383 (5)	O6—H6A	0.82
C1—C6	1.392 (5)	C42—C43	1.497 (6)
C1—C2	1.482 (5)	C43—C48	1.370 (6)
C2—C10	1.375 (6)	C43—C44	1.374 (6)
C2—C7	1.385 (5)	C44—C45	1.374 (7)
C3—C4	1.385 (5)	C44—H44	0.93
C3—H3	0.93	C45—C46	1.359 (7)
C4—H4	0.93	C45—H45	0.93
C5—C6	1.376 (5)	C46—C47	1.362 (7)
C5—H5	0.93	C46—H46	0.93
C6—H6	0.93	C47—C48	1.365 (6)
C7—C8	1.367 (6)	C47—H47	0.93
C7—H7	0.93	C48—H48	0.93
C8—H8	0.93	O7—C49	1.330 (5)
C9—C10	1.388 (6)	O7—H7A	0.82
C9—H9	0.93	O8—C49	1.198 (5)
C10—H10	0.93	C49—C50	1.482 (6)
C11—C13	1.358 (5)	C50—C55	1.366 (6)
C11—C16	1.359 (5)	C50—C51	1.390 (6)
C11—C12	1.482 (5)	C51—C52	1.373 (6)
C12—C20	1.359 (6)	C51—H51	0.93
C12—C17	1.391 (6)	C52—C53	1.356 (8)
C13—C14	1.368 (6)	C52—H52	0.93
C13—H13	0.93	C53—C54	1.368 (8)
C14—H14	0.93	C53—H53	0.93
C15—C16	1.367 (6)	C54—C55	1.394 (7)
C15—H15	0.93	C54—H54	0.93
C16—H16	0.93	C55—H55	0.93
C17—C18	1.375 (6)	O9—C35	1.316 (5)
C17—H17	0.93	O9—H9A	0.82
C18—H18	0.93	O10—C35	1.196 (5)
C19—C20	1.427 (7)	C35—C36	1.507 (6)
C19—H19	0.93	C36—C41	1.350 (6)
C20—H20	0.93	C36—C37	1.362 (6)
C21—C22	1.491 (5)	C37—C38	1.385 (7)
C22—C27	1.368 (5)	C37—H37	0.93
C22—C23	1.382 (5)	C38—C39	1.352 (7)
C23—C24	1.383 (6)	C38—H38	0.93
C23—H23	0.93	C39—C40	1.372 (7)
C24—C25	1.358 (7)	C39—H39	0.93
C24—H24	0.93	C40—C41	1.377 (7)
C25—C26	1.346 (7)	C40—H40	0.93
C25—H25	0.93	C41—H41	0.93
			
O3—Co1—O4	111.24 (10)	C23—C24—H24	119.9
O3—Co1—N1	93.58 (11)	C26—C25—C24	120.2 (5)
O4—Co1—N1	93.96 (11)	C26—C25—H25	119.9
O3—Co1—N3	87.57 (11)	C24—C25—H25	119.9
O4—Co1—N3	89.12 (11)	C25—C26—C27	120.7 (5)
N1—Co1—N3	176.07 (11)	C25—C26—H26	119.7
O3—Co1—O1	96.72 (10)	C27—C26—H26	119.7
O4—Co1—O1	151.17 (10)	C22—C27—C26	120.0 (5)
N1—Co1—O1	91.07 (10)	C22—C27—H27	120
N3—Co1—O1	85.06 (10)	C26—C27—H27	120
O3—Co1—O2	155.49 (10)	O3—C28—O4^i^	124.4 (3)
O4—Co1—O2	91.70 (10)	O3—C28—C29	118.8 (3)
N1—Co1—O2	93.29 (10)	O4^i^—C28—C29	116.8 (3)
N3—Co1—O2	84.17 (10)	C30—C29—C34	119.0 (3)
O1—Co1—O2	59.65 (9)	C30—C29—C28	120.1 (3)
C21—O1—Co1	90.4 (2)	C34—C29—C28	120.9 (3)
C21—O2—Co1	89.0 (2)	C31—C30—C29	120.8 (4)
C28—O3—Co1	157.9 (2)	C31—C30—H30	119.6
C28^i^—O4—Co1	133.3 (2)	C29—C30—H30	119.6
C14—N1—C15	114.3 (3)	C32—C31—C30	119.3 (4)
C14—N1—Co1	122.7 (3)	C32—C31—H31	120.3
C15—N1—Co1	122.8 (3)	C30—C31—H31	120.3
C19—N2—C18	118.2 (5)	C33—C32—C31	121.1 (4)
C4—N3—C5	116.6 (3)	C33—C32—H32	119.5
C4—N3—Co1	121.2 (2)	C31—C32—H32	119.5
C5—N3—Co1	122.2 (2)	C32—C33—C34	120.1 (4)
C9—N5—C8	117.2 (4)	C32—C33—H33	119.9
C3—C1—C6	116.7 (3)	C34—C33—H33	119.9
C3—C1—C2	121.9 (3)	C33—C34—C29	119.7 (4)
C6—C1—C2	121.3 (3)	C33—C34—H34	120.2
C10—C2—C7	116.9 (4)	C29—C34—H34	120.2
C10—C2—C1	122.8 (4)	C42—O6—H6A	109.6
C7—C2—C1	120.3 (4)	O5—C42—O6	123.4 (5)
C1—C3—C4	119.5 (4)	O5—C42—C43	124.3 (6)
C1—C3—H3	120.2	O6—C42—C43	112.3 (5)
C4—C3—H3	120.2	C48—C43—C44	119.0 (4)
N3—C4—C3	123.9 (4)	C48—C43—C42	122.9 (5)
N3—C4—H4	118	C44—C43—C42	118.1 (5)
C3—C4—H4	118	C43—C44—C45	120.3 (5)
N3—C5—C6	123.6 (3)	C43—C44—H44	119.9
N3—C5—H5	118.2	C45—C44—H44	119.9
C6—C5—H5	118.2	C46—C45—C44	120.1 (5)
C5—C6—C1	119.7 (3)	C46—C45—H45	120
C5—C6—H6	120.2	C44—C45—H45	120
C1—C6—H6	120.2	C45—C46—C47	119.8 (5)
C8—C7—C2	119.5 (5)	C45—C46—H46	120.1
C8—C7—H7	120.3	C47—C46—H46	120.1
C2—C7—H7	120.3	C46—C47—C48	120.5 (5)
N5—C8—C7	123.8 (5)	C46—C47—H47	119.8
N5—C8—H8	118.1	C48—C47—H47	119.8
C7—C8—H8	118.1	C47—C48—C43	120.3 (5)
N5—C9—C10	122.9 (5)	C47—C48—H48	119.8
N5—C9—H9	118.5	C43—C48—H48	119.8
C10—C9—H9	118.5	C49—O7—H7A	109.3
C2—C10—C9	119.7 (4)	O8—C49—O7	122.7 (4)
C2—C10—H10	120.2	O8—C49—C50	124.7 (5)
C9—C10—H10	120.2	O7—C49—C50	112.5 (5)
C13—C11—C16	115.6 (4)	C55—C50—C51	119.5 (4)
C13—C11—C12	122.9 (4)	C55—C50—C49	122.2 (5)
C16—C11—C12	121.5 (4)	C51—C50—C49	118.3 (4)
C20—C12—C17	116.1 (4)	C52—C51—C50	120.2 (5)
C20—C12—C11	123.2 (4)	C52—C51—H51	119.9
C17—C12—C11	120.7 (4)	C50—C51—H51	119.9
C11—C13—C14	120.8 (4)	C53—C52—C51	119.9 (5)
C11—C13—H13	119.6	C53—C52—H52	120.1
C14—C13—H13	119.6	C51—C52—H52	120.1
N1—C14—C13	124.3 (4)	C52—C53—C54	121.1 (5)
N1—C14—H14	117.9	C52—C53—H53	119.5
C13—C14—H14	117.9	C54—C53—H53	119.5
N1—C15—C16	124.5 (4)	C53—C54—C55	119.4 (6)
N1—C15—H15	117.8	C53—C54—H54	120.3
C16—C15—H15	117.8	C55—C54—H54	120.3
C11—C16—C15	120.4 (4)	C50—C55—C54	119.9 (5)
C11—C16—H16	119.8	C50—C55—H55	120
C15—C16—H16	119.8	C54—C55—H55	120
C18—C17—C12	120.1 (5)	C35—O9—H9A	109.7
C18—C17—H17	119.9	O10—C35—O9	124.4 (5)
C12—C17—H17	119.9	O10—C35—C36	123.1 (5)
N2—C18—C17	123.3 (5)	O9—C35—C36	112.5 (4)
N2—C18—H18	118.3	C41—C36—C37	119.1 (4)
C17—C18—H18	118.3	C41—C36—C35	123.2 (5)
N2—C19—C20	122.6 (5)	C37—C36—C35	117.7 (4)
N2—C19—H19	118.7	C36—C37—C38	120.9 (5)
C20—C19—H19	118.7	C36—C37—H37	119.5
C12—C20—C19	119.6 (5)	C38—C37—H37	119.5
C12—C20—H20	120.2	C39—C38—C37	119.3 (6)
C19—C20—H20	120.2	C39—C38—H38	120.3
O2—C21—O1	120.0 (3)	C37—C38—H38	120.3
O2—C21—C22	120.3 (3)	C38—C39—C40	120.2 (5)
O1—C21—C22	119.7 (4)	C38—C39—H39	119.9
C27—C22—C23	119.0 (4)	C40—C39—H39	119.9
C27—C22—C21	120.6 (4)	C39—C40—C41	119.4 (5)
C23—C22—C21	120.3 (4)	C39—C40—H40	120.3
C22—C23—C24	119.9 (4)	C41—C40—H40	120.3
C22—C23—H23	120	C36—C41—C40	121.0 (5)
C24—C23—H23	120	C36—C41—H41	119.5
C25—C24—C23	120.2 (5)	C40—C41—H41	119.5
C25—C24—H24	119.9		
			
O3—Co1—O1—C21	167.4 (2)	C11—C12—C17—C18	179.9 (4)
O4—Co1—O1—C21	1.4 (3)	C19—N2—C18—C17	2.2 (9)
N1—Co1—O1—C21	−98.8 (2)	C12—C17—C18—N2	−1.2 (9)
N3—Co1—O1—C21	80.5 (2)	C18—N2—C19—C20	−1.3 (8)
O2—Co1—O1—C21	−5.7 (2)	C17—C12—C20—C19	1.6 (7)
O3—Co1—O2—C21	−11.0 (4)	C11—C12—C20—C19	−179.1 (4)
O4—Co1—O2—C21	−170.8 (2)	N2—C19—C20—C12	−0.6 (8)
N1—Co1—O2—C21	95.1 (2)	Co1—O2—C21—O1	−9.8 (3)
N3—Co1—O2—C21	−81.9 (2)	Co1—O2—C21—C22	168.1 (3)
O1—Co1—O2—C21	5.8 (2)	Co1—O1—C21—O2	10.0 (3)
O4—Co1—O3—C28	−69.3 (7)	Co1—O1—C21—C22	−167.9 (3)
N1—Co1—O3—C28	26.4 (7)	O2—C21—C22—C27	3.6 (6)
N3—Co1—O3—C28	−157.4 (7)	O1—C21—C22—C27	−178.4 (4)
O1—Co1—O3—C28	117.9 (7)	O2—C21—C22—C23	−173.8 (4)
O2—Co1—O3—C28	132.4 (6)	O1—C21—C22—C23	4.2 (6)
O3—Co1—O4—C28^i^	18.2 (3)	C27—C22—C23—C24	0.5 (7)
N1—Co1—O4—C28^i^	−77.2 (3)	C21—C22—C23—C24	177.9 (4)
N3—Co1—O4—C28^i^	105.2 (3)	C22—C23—C24—C25	−0.2 (8)
O1—Co1—O4—C28^i^	−176.7 (3)	C23—C24—C25—C26	−0.1 (10)
O2—Co1—O4—C28^i^	−170.6 (3)	C24—C25—C26—C27	0.2 (11)
O3—Co1—N1—C14	−23.0 (3)	C23—C22—C27—C26	−0.5 (8)
O4—Co1—N1—C14	88.6 (3)	C21—C22—C27—C26	−177.9 (5)
O1—Co1—N1—C14	−119.8 (3)	C25—C26—C27—C22	0.2 (10)
O2—Co1—N1—C14	−179.4 (3)	Co1—O3—C28—O4^i^	65.7 (8)
O3—Co1—N1—C15	152.1 (3)	Co1—O3—C28—C29	−114.8 (6)
O4—Co1—N1—C15	−96.3 (3)	O3—C28—C29—C30	175.8 (3)
O1—Co1—N1—C15	55.3 (3)	O4^i^—C28—C29—C30	−4.7 (5)
O2—Co1—N1—C15	−4.3 (3)	O3—C28—C29—C34	−4.7 (5)
O3—Co1—N3—C4	−41.7 (3)	O4^i^—C28—C29—C34	174.9 (3)
O4—Co1—N3—C4	−153.0 (3)	C34—C29—C30—C31	−0.6 (6)
O1—Co1—N3—C4	55.3 (3)	C28—C29—C30—C31	179.0 (4)
O2—Co1—N3—C4	115.2 (3)	C29—C30—C31—C32	−0.6 (7)
O3—Co1—N3—C5	140.8 (3)	C30—C31—C32—C33	0.9 (8)
O4—Co1—N3—C5	29.5 (3)	C31—C32—C33—C34	−0.2 (8)
O1—Co1—N3—C5	−122.3 (3)	C32—C33—C34—C29	−1.0 (7)
O2—Co1—N3—C5	−62.3 (3)	C30—C29—C34—C33	1.3 (6)
C3—C1—C2—C10	−160.3 (4)	C28—C29—C34—C33	−178.2 (4)
C6—C1—C2—C10	22.2 (6)	O5—C42—C43—C48	−166.7 (5)
C3—C1—C2—C7	21.8 (6)	O6—C42—C43—C48	11.4 (6)
C6—C1—C2—C7	−155.8 (4)	O5—C42—C43—C44	12.2 (7)
C6—C1—C3—C4	1.6 (6)	O6—C42—C43—C44	−169.6 (4)
C2—C1—C3—C4	−176.0 (4)	C48—C43—C44—C45	−0.9 (7)
C5—N3—C4—C3	−1.5 (6)	C42—C43—C44—C45	−179.9 (4)
Co1—N3—C4—C3	−179.2 (3)	C43—C44—C45—C46	0.3 (8)
C1—C3—C4—N3	0.0 (6)	C44—C45—C46—C47	0.8 (9)
C4—N3—C5—C6	1.4 (5)	C45—C46—C47—C48	−1.3 (9)
Co1—N3—C5—C6	179.0 (3)	C46—C47—C48—C43	0.7 (8)
N3—C5—C6—C1	0.3 (5)	C44—C43—C48—C47	0.4 (7)
C3—C1—C6—C5	−1.8 (5)	C42—C43—C48—C47	179.3 (4)
C2—C1—C6—C5	175.9 (3)	O8—C49—C50—C55	−167.8 (4)
C10—C2—C7—C8	−0.8 (7)	O7—C49—C50—C55	12.5 (6)
C1—C2—C7—C8	177.3 (4)	O8—C49—C50—C51	12.8 (6)
C9—N5—C8—C7	0.0 (8)	O7—C49—C50—C51	−166.9 (4)
C2—C7—C8—N5	0.1 (8)	C55—C50—C51—C52	0.2 (6)
C8—N5—C9—C10	0.7 (8)	C49—C50—C51—C52	179.7 (4)
C7—C2—C10—C9	1.4 (7)	C50—C51—C52—C53	−0.1 (7)
C1—C2—C10—C9	−176.6 (4)	C51—C52—C53—C54	0.2 (9)
N5—C9—C10—C2	−1.5 (8)	C52—C53—C54—C55	−0.3 (9)
C13—C11—C12—C20	−25.2 (6)	C51—C50—C55—C54	−0.4 (7)
C16—C11—C12—C20	155.5 (5)	C49—C50—C55—C54	−179.8 (4)
C13—C11—C12—C17	154.0 (5)	C53—C54—C55—C50	0.4 (8)
C16—C11—C12—C17	−25.2 (6)	O10—C35—C36—C41	177.0 (5)
C16—C11—C13—C14	1.6 (7)	O9—C35—C36—C41	−3.8 (7)
C12—C11—C13—C14	−177.7 (4)	O10—C35—C36—C37	−3.2 (7)
C15—N1—C14—C13	−2.8 (7)	O9—C35—C36—C37	176.0 (5)
Co1—N1—C14—C13	172.6 (4)	C41—C36—C37—C38	0.8 (9)
C11—C13—C14—N1	1.3 (8)	C35—C36—C37—C38	−179.1 (5)
C14—N1—C15—C16	1.7 (7)	C36—C37—C38—C39	−1.1 (10)
Co1—N1—C15—C16	−173.8 (4)	C37—C38—C39—C40	1.1 (10)
C13—C11—C16—C15	−2.7 (7)	C38—C39—C40—C41	−0.8 (9)
C12—C11—C16—C15	176.6 (4)	C37—C36—C41—C40	−0.4 (8)
N1—C15—C16—C11	1.1 (8)	C35—C36—C41—C40	179.5 (5)
C20—C12—C17—C18	−0.8 (7)	C39—C40—C41—C36	0.4 (8)

Symmetry code: (i) −*x*, −*y*, −*z*+1.

### Hydrogen-bond geometry (Å, º)

**Table:**

*D*—H···*A*	*D*—H	H···*A*	*D*···*A*	*D*—H···*A*
O6—H6*A*···N2^ii^	0.82	1.91	2.726 (5)	177
O7—H7*A*···O1^iii^	0.82	1.86	2.656 (4)	164
O9—H9*A*···N5^iv^	0.82	1.91	2.732 (5)	176

Symmetry codes: (ii) *x*+1, *y*, *z*; (iii) −*x*, −*y*+1, −*z*+1; (iv) *x*−1, *y*+1, *z*−1.
